# *RNaseT2* knockout rats exhibit hippocampal neuropathology and deficits in memory

**DOI:** 10.1242/dmm.032631

**Published:** 2018-06-27

**Authors:** Kerstin W. Sinkevicius, Thomas R. Morrison, Praveen Kulkarni, Martha K. Caffrey Cagliostro, Sade Iriah, Samantha Malmberg, Julia Sabrick, Jennifer A. Honeycutt, Kim L. Askew, Malav Trivedi, Craig F. Ferris

**Affiliations:** 1 Preclinical Pharmacology, Alexion Pharmaceuticals, Lexington, MA 02421, USA; 2 Center for Translational Neuroimaging, Department of Psychology and Pharmaceutical Sciences, Northeastern University, Boston, MA 02115, USA; 3 Division of Developmental Neuroscience, Department of Psychiatry, Columbia University, New York, NY 10032, USA; 4 Department of Pharmaceutical Sciences, College of Pharmacy, Nova Southeastern University, Fort Lauderdale, FL 33314, USA

**Keywords:** Diffusion-weighted imaging, Magnetic resonance imaging, CRISPR/Cas9, Lysosomal storage disease, Object recognition, Glial fibrillary acidic protein

## Abstract

RNASET2 deficiency in humans is associated with infant cystic leukoencephalopathy, which causes psychomotor impairment, spasticity and epilepsy. A zebrafish mutant model suggests that loss of RNASET2 function leads to neurodegeneration due to the accumulation of non-degraded RNA in the lysosomes. The goal of this study was to characterize the first rodent model of RNASET2 deficiency. The brains of 3- and 12-month-old *RNaseT2* knockout rats were studied using multiple magnetic resonance imaging modalities and behavioral tests. While T1- and T2-weighted images of *RNaseT2* knockout rats exhibited no evidence of cystic lesions, the prefrontal cortex and hippocampal complex were enlarged in knockout animals. Diffusion-weighted imaging showed altered anisotropy and putative gray matter changes in the hippocampal complex of the *RNaseT2* knockout rats. Immunohistochemistry for glial fibrillary acidic protein (GFAP) showed the presence of hippocampal neuroinflammation. Decreased levels of lysosome-associated membrane protein 2 (LAMP2) and elevated acid phosphatase and β-N-acetylglucosaminidase (NAG) activities indicated that the *RNASET2* knockout rats likely had altered lysosomal function and potential defects in autophagy. Object recognition tests confirmed that *RNaseT2* knockout rats exhibited memory deficits. However, the Barnes maze, and balance beam and rotarod tests indicated there were no differences in spatial memory or motor impairments, respectively. Overall, patients with RNASET2 deficiency exhibited a more severe neurodegeneration phenotype than was observed in the *RNaseT2* knockout rats. However, the vulnerability of the knockout rat hippocampus as evidenced by neuroinflammation, altered lysosomal function and cognitive defects indicates that this is still a useful *in vivo* model to study RNASET2 function.

## Abstract (precis)

**Summary:** The authors characterize a rodent model of *RNaseT2* deficiency, which offers insight into the susceptibility of the hippocampus to early inflammation caused by lysosome impairment due to loss of *RNaseT2* function.

## INTRODUCTION

Ribonucleases (RNases) are ubiquitously expressed enzymes which catalyze the cleavage of RNA and function in a variety of cellular processes, including RNA degradation, RNA processing and maturation, viral defense, and RNA interference (RNAi). Transferase-type RNases hydrolyze single-stranded RNA to mononucleotides or oligonucleotides with a 3′-terminal phosphate via a 2′,3′-cyclic phosphate intermediate. These RNases are grouped into the RNase A, T1 and T2 families based on their base specificity, optimal pH and origin (Irie, 1999; Luhtala and Parker, 2010). Unlike other ribonuclease families, RNASET2 is optimally active in an acidic environment of pH4 to 5, which is consistent with its localization in lysosomes and vacuoles (Luhtala and Parker, 2010). Human RNASET2 may have several functional roles that are dependent on its ribonuclease activity, including scavenging extracellular nucleic acids for nutrients during cellular stress and recycling cytoplasmic RNAs that are delivered to lysosomes or vacuoles during autophagy (Luhtala and Parker, 2010).

RNASET2 deficiency has been described previously in patients and a zebrafish model. Mutations in the human *RNaseT2* gene resulting in the loss of a functional gene product are associated with cystic leukoencephalopathy that arises in infancy, characterized by cortical cysts, multifocal white matter lesions, and calcifications in the brain (Henneke et al., 2009). Affected patients exhibit psychomotor impairment, spasticity and epilepsy (Henneke et al., 2009). Mutant zebrafish deficient in RNASET2 activity develop increased levels of undigested ribosomal RNA (rRNA) within the lysosomes of neurons and neurodegeneration around sites of white matter lesions (Haud et al., 2011). These results suggest that loss of RNASET2 function leads to accumulation of non-degraded RNAs in the lysosomes. Accumulation of these rRNAs may impair lysosome function and indirectly inhibit macroautophagy, be cytotoxic and/or induce an innate immune response which leads to neurodegeneration (Haud et al., 2011). Loss of RNASET2 may therefore cause a lysosomal storage disorder.

The goal of this study was to develop and characterize the first rodent model of RNASET2 deficiency. Rats were selected for these studies because *RNaseT2* is a single-copy gene in rats but there are two RNaseT2-encoding paralogs in mice. Additional advantages to using rats over mice include a more translatable neurobiology, better established behavioral assays and higher resolution for brain imaging. *RNaseT2* knockout (KO) rats were generated with CRISPR/Cas9 technology.

Unlike patients with RNASET2 deficiency, cortical cysts, multifocal white matter lesions and calcifications were not observed in the brains of 3- and 12-month-old *RNaseT2* KO rats. However, the KO rats did have hippocampal morphology alterations, neuroinflammation, lysosomal dysfunction and cognitive defects, indicating that, although the knockout rat phenotype was not as severe as the symptomology observed in patients, the observed deficits were likely driven by similar underlying mechanisms.

## RESULTS

### Generation of *RNaseT2* knockout rats using CRISPR/Cas9 technology

Two pairs of CRISPR guide RNAs were designed to cleave together to delete all 9 exons of *RNaseT2* (Fig. S1A). The CRISPR guide RNA and *Cas9* mRNA mixtures were microinjected into the pronuclei of fertilized embryos of Sprague-Dawley rats and transferred to pseudopregnant female rats. Founder lines were screened for the ∼17 kB genomic DNA deletion by PCR and Sanger sequencing (Fig. S1B). Lines 11, 12 and 19 were backcrossed to wild-type (WT) animals to generate F1 heterozygous animals. Brain *RNaseT2* transcript levels confirmed successful knockout of *RNaseT2* in these homozygous lines (Fig. S1C). The 12-colony founder line was expanded and used for all subsequent studies. WT, heterozygous and homozygous (KO) rats were born in the expected Mendelian ratio, and homozygous *RNaseT2* KO rats were viable and fertile.

### Characterization of 3-month-old *RNaseT2* knockout rats

The phenotype of 3-month-old *RNaseT2* KO rats was determined using brain histopathology, magnetic resonance imaging (MRI), immunohistochemistry, lysosomal analysis and behavioral tests. Full necropsies, in which 39 tissues were collected and processed, were performed on 2 WT and 3 *RNaseT2* KO female rats (see Materials and Methods for details). Brains and male reproductive tissues from 3 WT and 3 *RNaseT2* KO males were also collected. No abnormalities were found in any tissues by histopathological analysis (data not shown).

**Fig. 1.**
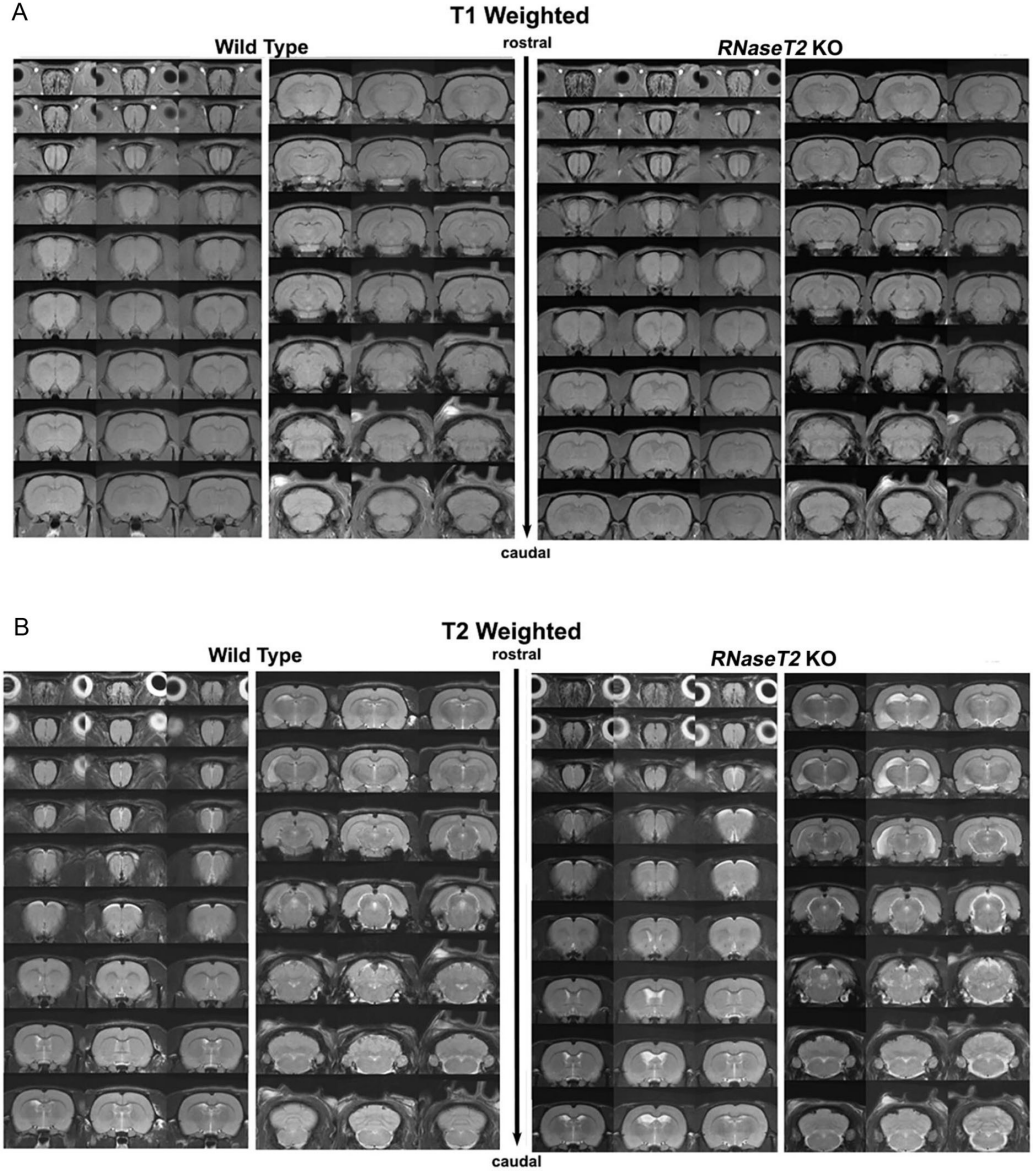
**T1- and T2-weighted brain images of 3-month-old wild-type (WT) and *RNaseT2* KO rats.** Representative (A) T1- and (B) T2-weighted brain images of contiguous, 1 mm thick, axial sections from three examples of neuroanatomy from WT (*n*=5) and *RNaseT2* KO (*n*=6) rats. Each column is a separate subject.

Next, 3-month-old WT and *RNaseT2* KO male rats were scanned using T1-, T2- and diffusion-weighted imaging (DWI) protocols. The general morphology of the T1-weighted (Fig. 1A) and T2-weighted (Fig. 1B) images shown as 2D anatomical sections spanning the entire brain were the same between the 2 genotypes. There were no white matter tract abnormalities, cysts or hyper-intensities in the frontal or temporal regions or caudate/putamen of the *RNaseT2* KO rats.

**Fig. 2.**
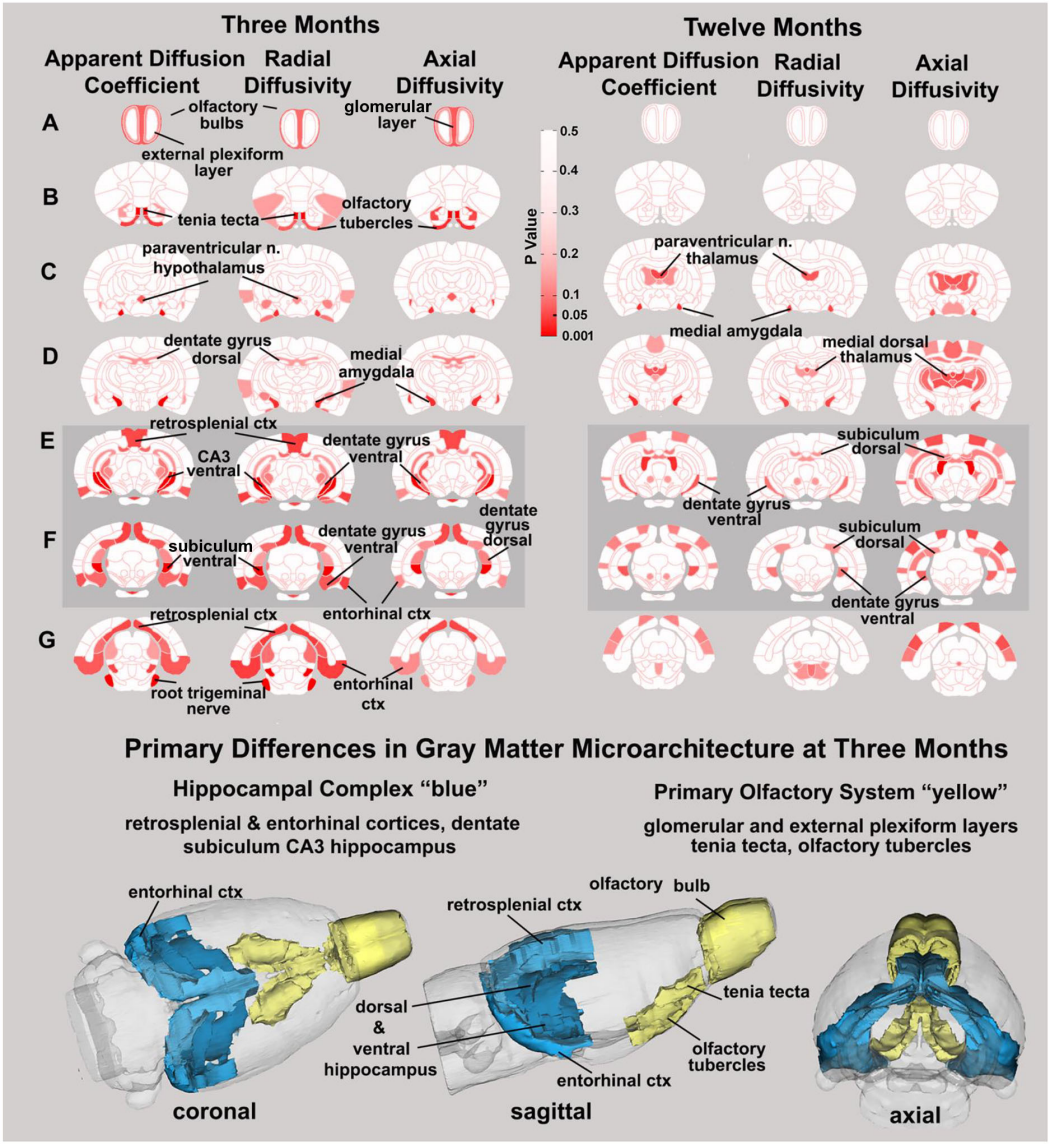
**Probability maps for indices of anisotropy comparing WT and *RNaseT2* KO rats at 3 and 12 months of age.** Diffusion-weighted imaging 2D probability maps with quantitative anisotropy highlighting the brain areas (pink/red) that are significantly different between WT and *RNaseT2* KO rats at 3 months (*n*=5 for each genotype) and 12 months (*n*=6 for each genotype) of age. The brain areas that were significantly different across all indices of anisotropy, apparent diffusion coefficient (ADC), radial diffusivity (RD) and axial diffusivity (AD) at 3 months of age were associated with two major neural circuits, the hippocampal complex and the primary olfactory system. The 3D representations of these areas are presented below in different orthogonal directions. ctx, cortex; n., nucleus.

DWI shows changes in anisotropy, a sign of altered gray matter microarchitecture and possible neurodegeneration. DWI combined with computational analysis using a 3D segmented rat MRI atlas with 174 brain regions revealed differences in indices of anisotropy between numerous brain regions of the WT and *RNaseT2* KO rats. Probability maps highlighting the significant differences between the 3-month-old WT and *RNaseT2* KO rats identified several brain areas, including the glomerular layer of olfactory bulb, tenia tecta, medial amygdala, dentate and subiculum of the hippocampus, and retrosplenial and entorhinal cortices (Fig. 2 and Tables S1, S2). At 3 months of age, the differences were primarily associated with the primary olfactory system highlighted in yellow in the 3D representations and the hippocampal complex highlighted in blue (Fig. 2). These highlighted measurements were significantly increased in the *RNaseT2* KO rats compared to the WT animals.

**Fig. 3.**
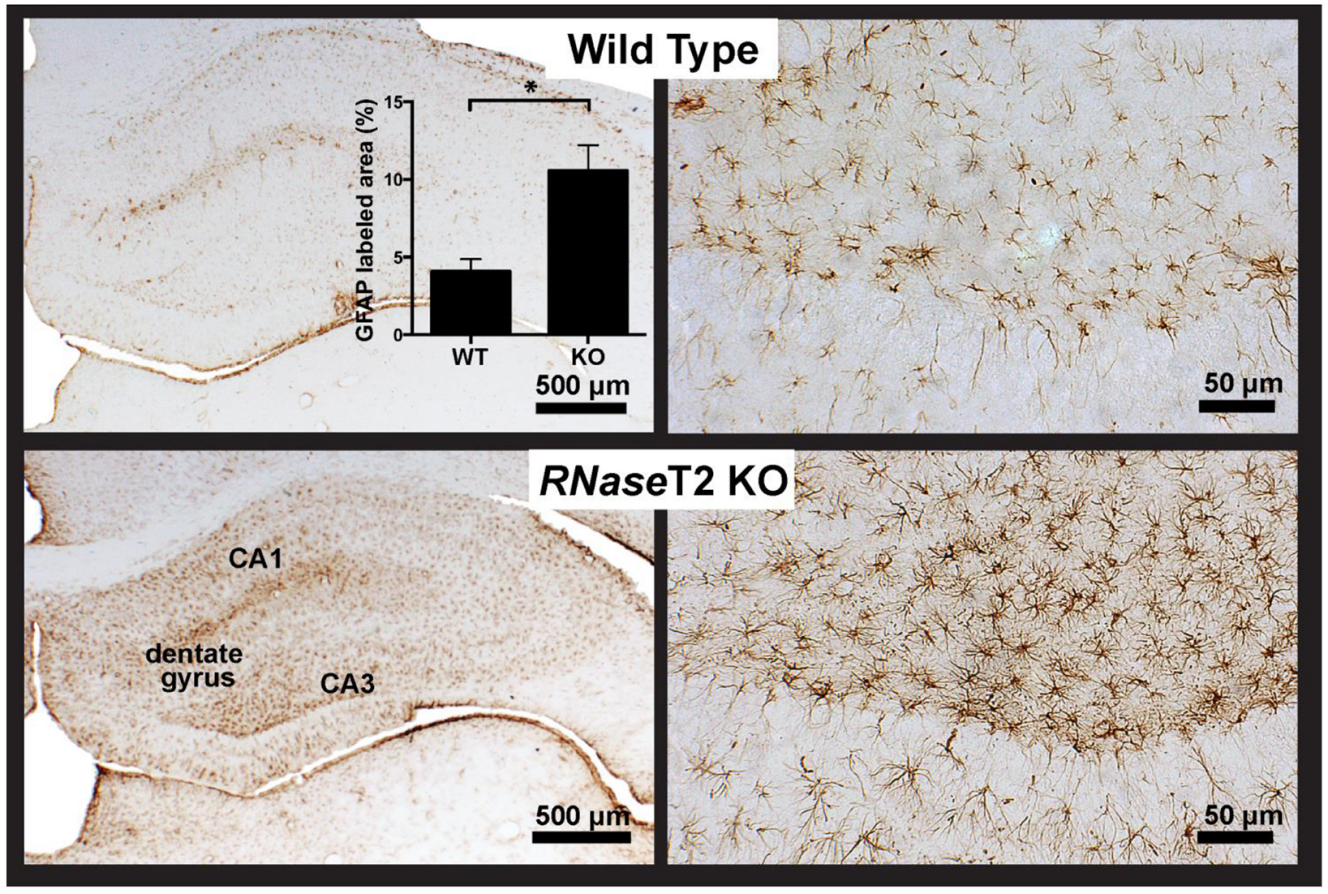
**Hippocampal neuroinflammation GFAP analysis of 3-month-old WT and *RNaseT2* KO rats.** Representative immunohistochemistry of GFAP staining in the dorsal hippocampus of 3-month-old WT (top) and *RNaseT2* KO (bottom) rats at low (4×; left images) and high (20×; right images) magnification. The insert (top left) shows a bar graph of the mean percentage of area showing immunostaining for GFAP in WT (*n*=5) and KO (*n*=4) rats. *P<0.05 with an unpaired two-tailed Student's *t*-test.

Glial fibrillary acidic protein (GFAP) immunohistochemistry showed that the number of reactive astrocytes, a sign of neuroinflammation (Eng and Ghirnikar, 1994; Eng et al., 1992), were significantly higher in the hippocampus of the *RNaseT2* KO rats compared to the WT animals (Fig. 3). This corresponded to the DWI results and demonstrates that the hippocampi of the 3-month-old *RNaseT2* KO rats show putative indications of neuroinflammation.

**Fig. 4.**
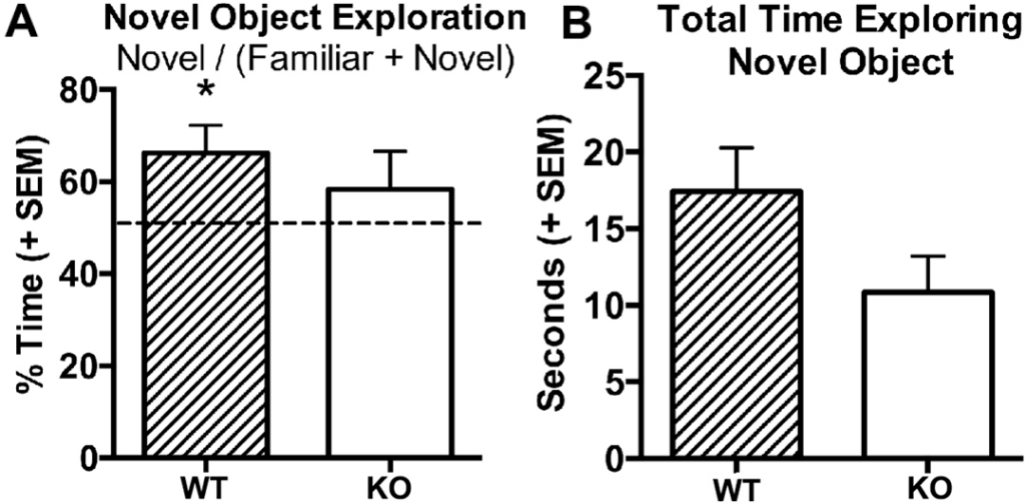
**Object recognition memory test comparing 3-month-old WT and *RNaseT2* KO rats.** (A) Mean percentage of time spent exploring the novel object during the familiar and novel stages of a novel object recognition (NOR) test. (B) Total amount of time during the NOR test that WT (*n*=10) and *RNaseT2* KO (*n*=9) rats spent exploring the novel object. Vertical bars denote s.e.m. *P<0.05 for single-sample *t*-test that was used to determine if preference for the novel object exceeded or fell below chance (theoretical mean set at 0.5 or 50%).

To determine whether the hippocampal neuroinflammation observed in the *RNaseT2* KO rats was causing a cognitive defect, a novel-object-recognition memory test was performed. WT (*t*_9_=2.71, *P=*0.02), but not *RNaseT2* KO rats (*t*_8_=1.02, *P=*0.34), had a significantly greater preference for the novel object, beyond chance (>50%), during the novel phase when tested at 3 months of age (Fig. 4A). Although there was no difference between the genotypes for the novel-object-recognition index (*t*_17_=0.78, *P=*0.44) or total time exploring the novel object (*t*_17_=1.76, *P=*0.09) during the novel phase, there was a trend that the *RNaseT2* KO rats spent less time exploring the novel object (Fig. 4B). These results indicated that the 3-month-old *RNaseT2* KO rats may have a defect in episodic learning and memory-related stimulus recognition.

### Characterization of 12-month-old *RNaseT2* KO rats

To determine whether the neuroradiological and behavioral endophenotypes of *RNaseT2* KO rats progressed with age, 12-month-old animals were characterized. Similarly to what was observed in the T1- and T2-weighted brain MRIs of the 3-month-old animals, there was no evidence of abnormalities in the 12-month-old *RNaseT2* KO rats (data not shown). The probability maps for differences in indices of anisotropy showed changes in the hippocampal complex as observed in the 3-month-old *RNaseT2* KO rats, but not the other brain regions (Fig. 2).

**Fig. 5.**
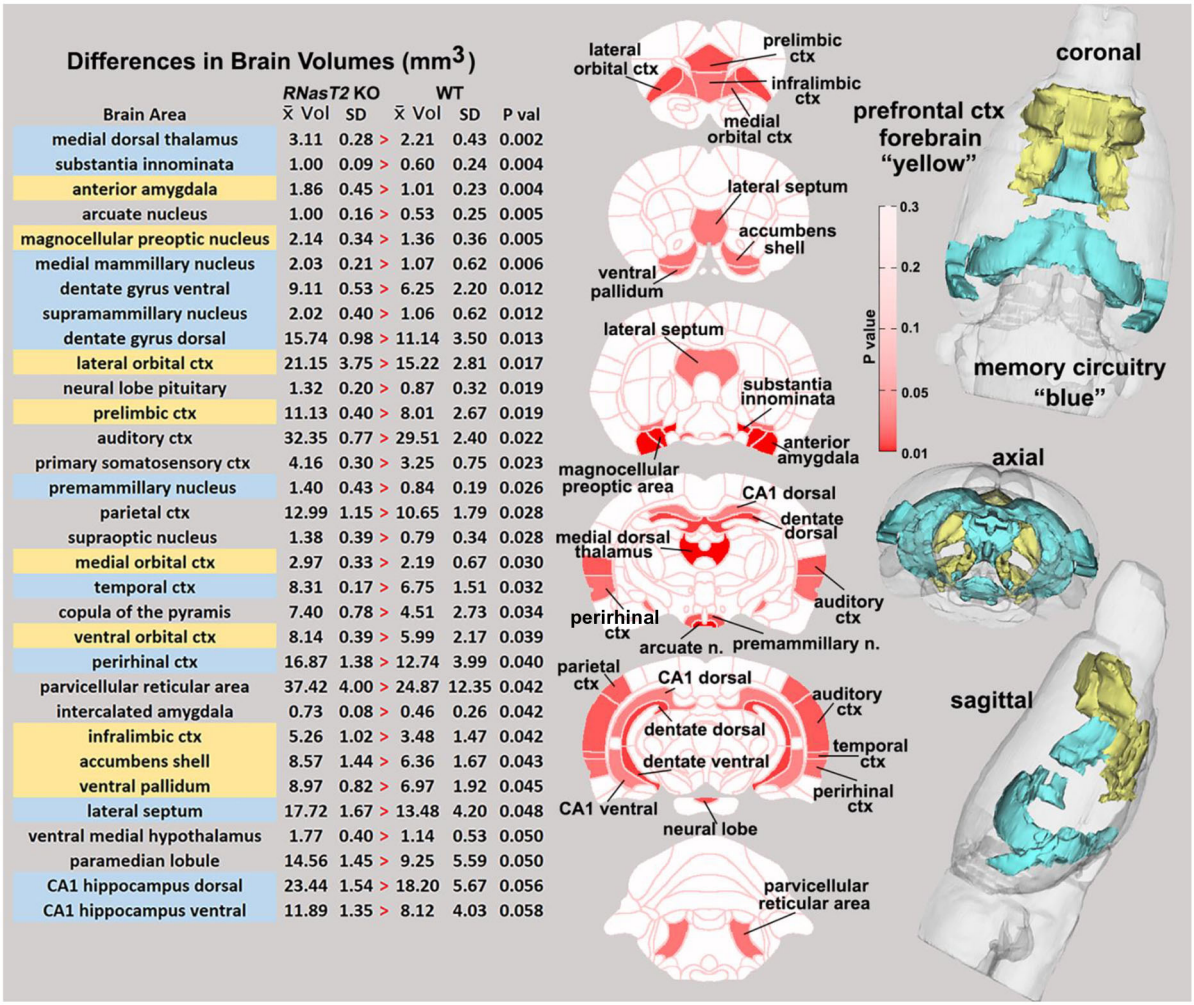
**Quantitative brain volumes of 12-month-old WT and *RNaseT2* KO rats.** 2D probability maps of quantitative volumetric measure of brain regions highlighting the brain areas (pink/red) that are significantly different in volume between 12-month-old WT (*n*=6) and *RNaseT2* KO (*n*=6) rats. The table shows the mean 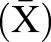 and s.d. for those areas that were significantly different from the 174 regions defined in the segmented MRI rat atlas. Note that, in all cases, the *RNaseT2* KO brains had larger volumes. These areas are ranked in order of their significance. Many of these areas were associated with the prefrontal cortex and ventral forebrain (highlighted in yellow) and neural circuitry involved in learning and memory (highlighted in blue). The 3D representations of these associated areas are shown to the right in different orthogonal directions. ctx, cortex; n., nucleus.

Quantitative volumetric analysis using the 3D segmented rat MRI atlas with 174 brain regions revealed enlarged brain volumes in *RNaseT2* KO rats as compared to WT animals in specific brain areas (Fig. 5 and Table S3). The affected brain areas tended to cluster in the prefrontal cortex (e.g. prelimbic, infralimbic, medial orbital, lateral orbital cortices) and ventral striatum (e.g. ventral pallidum, accumbens shell). Circuitry associated with learning and memory was clearly affected in *RNaseT2* KO rats, with increased volumes in the medial dorsal thalamus, substantia innominate, mammillary nuclei, temporal cortex, septum and hippocampal complex (all highlighted in blue  in Fig. 5).

**Fig. 6.**
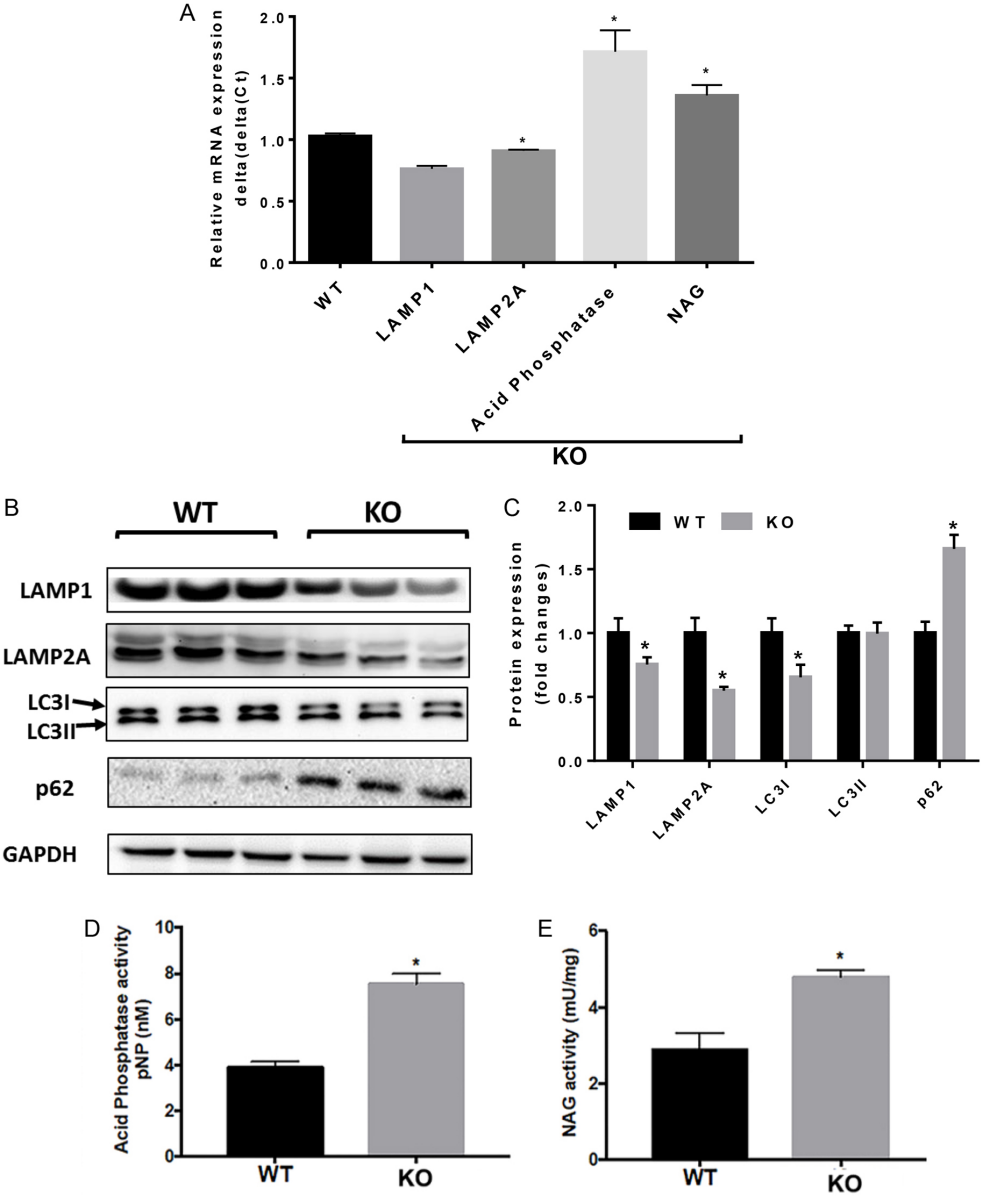
**Brain lysosome analysis of 12-month-old WT and *RNaseT2* KO rats.** (A) qPCR analysis of hippocampal LAMP1, LAMP2A, acid phosphatase and NAG levels of WT (*n*=3) and *RNaseT2* KO (*n*=3) rats. (B) Western blot analysis of hippocampal lysosomal (LAMP1 and LAMP2A) and autophagy (LC3I, LC3II and p62) markers of WT (*n*=3) and *RNaseT2* KO (*n*=3) rats. (C) Quantification of western blots in B using ImageJ; GAPDH was used as an internal loading control and normalized to WT. (D) Acid phosphatase enzyme activity of WT (*n*=3) and *RNaseT2* KO (*n*=3) rats. (E) NAG enzyme activity of WT (*n*=3) and *RNaseT2* KO (*n*=3) rats. Vertical bars denote s.e.m. *P<0.05 with an unpaired two-tailed Student's *t*-test.

Next, hippocampal sections from 12-month-old WT and *RNaseT2* KO rats were analyzed for lysosome integrity. Lysosome-associated membrane proteins 1 and 2 (LAMP1 and LAMP2) make up about 50% of lysosomal membrane glycoproteins and are highly important for lysosomal functions (Eskelinen, 2006). *LAMP2A* transcript levels were significantly lower in the *RNaseT2* KO animals as compared to WT controls (Fig. 6A). Although there was a trend that *LAMP1* mRNA levels were also lower, this difference was not statistically significant (Fig. 6A). However, this trend had consequences since the hippocampal protein levels of both *LAMP1* and *LAMP2A* were significantly lower in the KO rats compared to WT animals (Fig. 6B,C). In addition, the mRNA levels of lysosomal enzymes involved in hydrolysis of phosphates and degradation of lysosomal glycoproteins, acid phosphatase and β-N-acetylglucosaminidase (NAG), respectively, were significantly elevated in the *RNaseT2* KO rats as compared to the WT animals (Fig. 6A). These elevated mRNA levels corresponded to higher levels of enzyme activity for both lysosomal enzymes (Fig. 6D,E). Since lysosomal functions are directly associated with autophagy, we also measured the expression of autophagy-related proteins. Protein levels of p62, a direct regulator of autophagolysosome formation, were elevated in the *RNaseT2* KO rats (Fig. 6B,C). Protein levels of LC3I and LC3II, other important autophagy markers, were also measured. Levels of LC3I, but not LC3II, were lower in the KO animals compared to WT (Fig. 6B,C), indicating impaired autophagy. Overall, these results suggest that the *RNaseT2* KO animals may have altered hippocampal lysosomal function and autophagy.

**Fig. 7.**
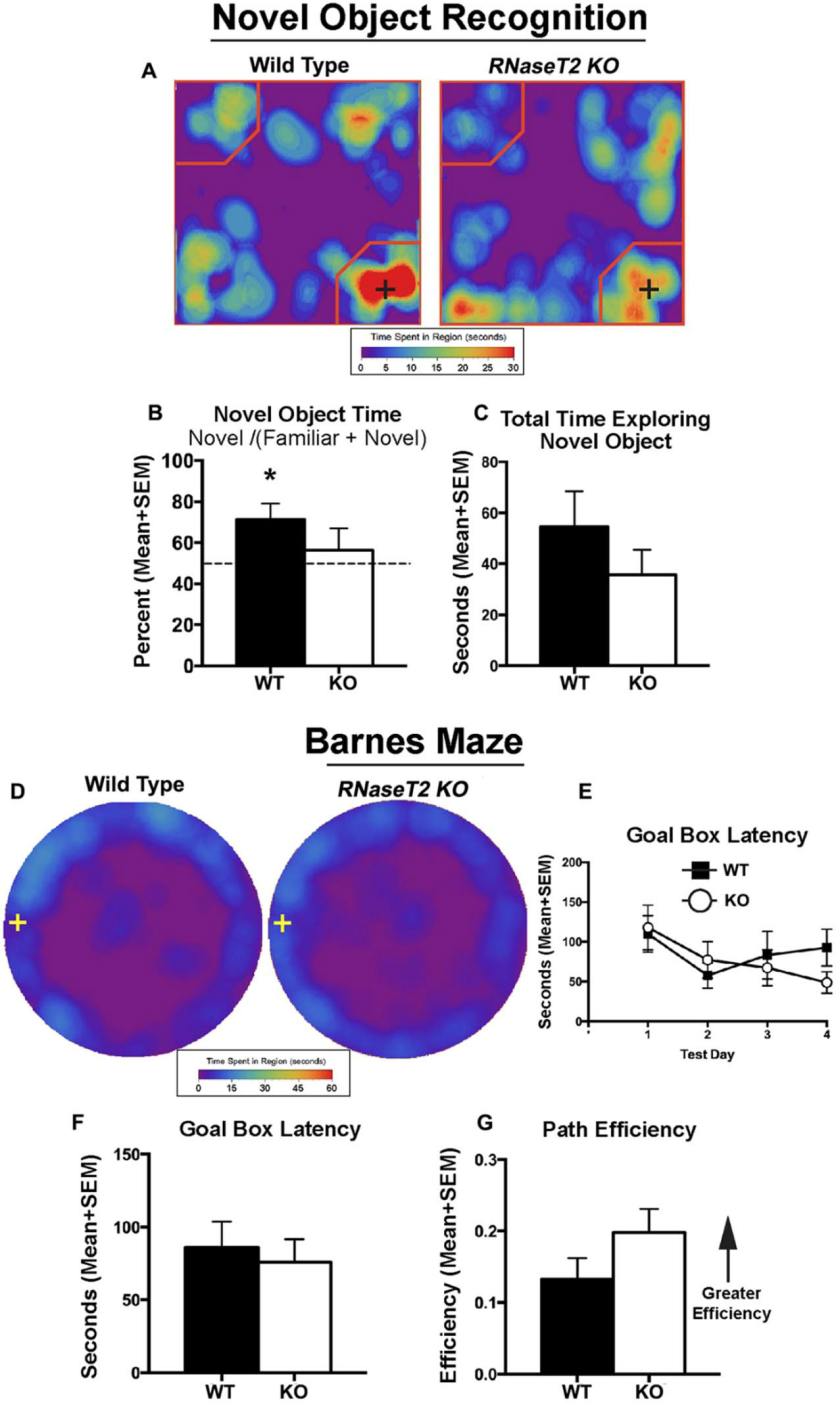
**Cognitive behavior of 12-month-old WT and *RNaseT2* KO rats.** Novel object recognition (NOR): (A) heat maps for the novel phase of the NOR memory test. Only slight qualitative differences were evident between groups on the heat maps as indicated by the presence of red on the lower right corner of the maps. The black cross indicates the location of the novel object. (B) Mean percentage of time spent exploring the novel object during the familiar and novel stages of a NOR test for WT (*n*=6) and *RNaseT2* KO (*n*=12) rats. (C) Total time spent exploring the novel object. Vertical bars denote s.e.m. *P<0.05 for single-sample *t*-test. Barnes maze: (D) qualitative data from averaged heat maps showing an overhead view of movement patterns on the Barnes maze (goal box is indicated by the cross on the left side) for WT (*n*=6) and *RNaseT2* KO (*n*=11) rats. (E) Goal box latency across days. (F) Goal box latency collapsed across days. (G) Path efficiency across all days. Vertical bars denote s.e.m.

Novel-object-recognition and Barnes maze tests were performed to determine whether the deficit in cognition observed at 3 months progressed with age. The results from the novel-object-recognition test of the 12-month-old *RNaseT2* KO rats were similar to the 3-month-old animals. WT rats (*t*_5_=2.751, *P=*0.04), but not *RNaseT2* KO rats (*t*_10_=0.59, *P=*0.57), had a significantly greater preference for the novel object, beyond chance (>50%), during the novel phase (Fig. 7A,B). However, there was no difference between the genotypes for novel-object-recognition index (*t*_15_=0.95, *P=*0.35), total time exploring the novel object (*t*_15_=1.11, *P=*0.29) or the percentage of novel object encounters during the novel phase (*t*_15_=0.70, *P=*0.50) (Fig. 7B,C). The Barnes maze test, which measures spatial learning and memory, showed a significant main effect of testing day on goal box latency across days (*F*_3,39_=3.9, *P*=0.02), but no significant difference between WT and *RNaseT2* KO rats (*F*_1,13_=0.09, *P*=0.77) (Fig. 7D,E). Both genotypes equally had significantly shorter latencies to enter the goal box on testing day 2 (*P*=0.005), 3 (*P*=0.01) and 4 (*P*=0.003) when compared to the first day of testing (Fig. 7E). Conversely, path efficiency across days remained relatively constant, showing no effect of test day (*F*_3,39_=0.78, *P*=0.51) or differences between genotypes (*F*_1,13_=0.91, *P*=0.51). When collapsed across days, there were no differences between genotypes for either goal box latency (*t*_15_=0.40, *P*=0.70) or path efficiency (*t*_15_=1.32, *P*=0.21) (Fig. 7F,G). Overall, these results indicate that the *RNaseT2* KO rats may have a defect in episodic learning and memory-related stimulus recognition, and that this deficit does not decline with age.

Motor behavior testing was also performed in 12-month-old animals since RNASET2-deficient patients exhibit psychomotor impairment, spasticity and epilepsy (Henneke et al., 2009). For balance beam testing, there were no significant differences between the genotypes for total foot faults (*t*_15_=0.40, *P*=0.69) or goal box latency (*t*_15_=0.48, *P*=0.63) (Table 1). Analysis of balance beam performance in terms of the widths of the 3 specific beam segments (i.e. wide, middle and thin) resulted in a significant main effect of segment thickness (*F*_2,32_=25.8, *P*<0.0001), with both genotypes showing a higher number of foot faults on the thinnest portion of the beam compared to either the widest, or middle, portion of the beam (*P*<0.0001 for both), but no significant difference between genotypes. Similarly, there was no difference between the genotypes for fall latency on the rotarod task (*t*_15_=0.18, *P*=0.86). These results show that the 12-month-old *RNaseT2* KO rats do not exhibit any motor impairments.

**Table 1.**
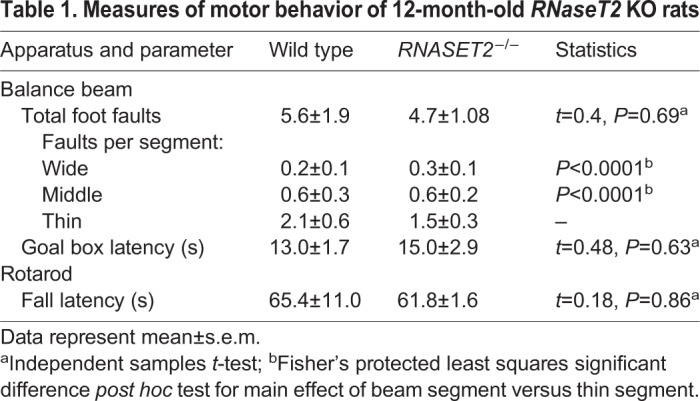
**Measures of motor behavior of 12-month-old *RNaseT2* KO rats**

## DISCUSSION

Studies of the rare autosomal recessive disorder cystic leukoencephalopathy without megalencephaly caused by *RNaseT2* mutations describe clear neuroradiological signs similar to congenital cytomegalovirus (CMV) brain infection (Henneke et al., 2009). In both cases, hallmark brain abnormalities identified through MRI are primarily localized to the frontal and temporal regions, and are characterized by multifocal white matter abnormalities, cysts and hyper-intensities. In the present study, multiple imaging modalities were used to identify the neuroradiological endophenotype(s) that defines the male *RNaseT2* KO rat. We were unable to observe any alterations in white matter integrity or microcysts/hyper-intensities in the brains of the 3- or 12-month-old *RNaseT2* KO rats with either T1- or T2-weighted high-resolution anatomical scans. However, quantitative volumetric measures taken from 174 brain regions revealed significant differences in discrete brain volumes between genotypes. *RNaseT2* KO animals had larger brain volumes primarily localized to the hippocampal complex and prefrontal cortex: areas roughly equivalent to the frontal and temporal regions in humans. These volumetric measures were only taken in 12-month-old rats, so we are uncertain whether these changes were already established at 3 months of age or if they developed over early adulthood.

Increased regional brain volumes have not been previously reported in *RNaseT2* animal models, to the best of our knowledge. Although the mechanism underlying this effect is unknown, it is possible that neuronal swelling contributed to the increased brain volumes in select regions, as swollen neurons can be found throughout the brains of patients with some lysosomal storage disorders. For example, gangliosides accumulate in the brains of patients with Niemann–Pick and Tay–Sachs disease, which causes neurons to swell and results in brain enlargement early in disease progression (Ferreira and Gahl, 2017). Similarly, in patients with unclassified leukoencephalopathies, post-mortem studies have shown swelling in the corpus callosum, thalamus, hypothalamus, basal ganglia, midbrain, pons, medulla, deep cerebral white matter and cerebellar white matter (Steenweg et al., 2012). Future studies that quantify brain gangliosides in *RNaseT2* KO rats will further determine the translatability to related conditions (e.g. GM1 and GM2 gangliosidosis) found in patients.

We also used DWI and quantitative anisotropy to follow alterations in gray matter microarchitecture from 3 to 12 months in *RNaseT2* KO rats as compared to WT animals. In a recent study, we used DWI and quantitative anisotropy to identify subtle changes in subcortical gray matter microarchitecture in rats following mild concussion (Kulkarni et al., 2015). The determinants of diffusion at a microscopic level are many as the microarchitecture of the brain parenchyma is composed of neurons and their axonal and dendritic fibers, glia, connective tissue, capillaries, and intracellular and extracellular water. It should be noted that microscopic axonal properties and general microarchitecture of a voxel are the key determinants of diffusion anisotropy and not myelination as originally posited (Moseley et al., 1991). In this study, analysis across 174 brain regions showed differences in indices of anisotropy primarily localized to the hippocampal complex and primary olfactory system. Interestingly, the changes were less pronounced at 12 months, suggesting that gray matter microarchitecture stabilizes with aging. Indeed, descriptions of the clinical signs and symptoms of cystic leukoencephalopathy without megalencephaly report greater severity in early childhood but little overt pathology in adulthood (Henneke et al., 2005; Tonduti et al., 2016). The only brain area that continued to show gray matter differences with aging in the *RNaseT2* KO rat was the hippocampus.

The volumetric neuroanatomy and DWI results showed a clear difference in the hippocampal complex between genotypes. This clear neuroradiological endophenotype suggested there would also be differences in cognitive behavior. Children with RNASET2 deficiency present with mental and motor retardation and oftentimes epilepsy (Henneke et al., 2005; Tonduti et al., 2016). There were no overt signs of motor dysfunction or seizure activity in 3- or 12-month-old *RNaseT2* KO rats. Both the 3- and 12-month-old *RNaseT2* KO cohorts showed deficits in object recognition memory. The 12-month-old KO cohort had both normal spatial memory and motor function. Although, we did not test the 3-month-old KO rats on the Barnes maze or using any motor apparatuses, DWI for these animals showed no changes in gray matter microarchitecture in neural circuits associated with motor control (e.g. basal ganglia, motor cortex or cerebellum).

As noted earlier, the clinical and radiological measures of RNASET2 deficiency and *in utero* CMV infection are almost identical, except the latter is noted for the presence of neuroinflammation as the key component of the pathophysiology (Cheeran et al., 2003, 2001). Given a recent study showing the radiological similarities between RNASET2 deficiency and CMV with Aicardi–Goutieres syndrome (AGS), another inherited disorder mimicking congenital infection with frontal and temporal cysts and pronounced neuroinflammation (Tonduti et al., 2016), we followed up on our imaging data by investigating the possibility that *RNaseT2* KO rats would present with GFAP-positive astrocytes in the hippocampus. Indeed, 3-month-old KO rats showed a significant elevation in GFAP-positive glia, and thus putative neuroinflammation, in the hippocampal complex as compared to WT animals.

It is likely that lysosome dysfunction played a key role in the hippocampal neuroinflammation that we observed. Indeed, it has been hypothesized that cystic leukoencephalopathy without megalencephaly, caused by loss of RNASET2 function, is a lysosomal storage disease, with accumulation of excess RNA eliciting an autoimmune and neuroinflammatory response akin to what is observed in CMV and AGS. Western blot analyses revealed that LAMP1 and LAMP2A protein levels were significantly lower in the hippocampus of *RNaseT2* KO rats compared to WT animals. Notably, these findings will need to be confirmed and validated using immunohistochemistry, which was attempted repeatedly but remained technically challenging. LAMP1 and LAMP2 are lysosome-associated membrane glycoproteins which act as receptors for proteins, adhesion (if expressed on the plasma membrane) and intercellular signal transduction (Eskelinen, 2006). Their main function is to protect the lysosomal membrane from proteolytic enzymes that are within the lysosome itself (as in autodigestion) (Eskelinen, 2006). The decreased protein levels of LAMP1 and LAMP2 observed in the *RNaseT2* KO rats could affect these functions, including lysosomal associated autophagy. Furthermore, such glycoproteins are degraded by the lysosomal enzymes, such as NAG, as well as acid phosphatases, and we observed that the activity of these enzymes was significantly elevated in the *RNaseT2* KO rats. In parallel, we observed changes in the levels of important autophagy regulators, including p62 and LC3, in the *RNaseT2* KO rats. These observed lysosomal dysfunctions could potentially contribute to autophagy induction, neuroinflammation, neuronal firing and synaptic activities (Kononenko, 2017).

In summary, our studies show that the *RNaseT2* KO rats exhibit hippocampal neuroinflammation and signs of cognitive impairment, which may be driven by lysosomal dysfunction. However, the animals did not present with the neuroradiological hallmarks, motor deficits or spontaneous seizure activity associated with RNASET2 deficiency in humans. Although the *RNaseT2* KO rat does not appear to completely model the human disease, perhaps due to protein compensation that is unique to the rat (e.g. by redundant RNAses), it may offer insights into the susceptibility of the hippocampus to early inflammation caused by lysosome impairment.

## MATERIALS AND METHODS

### *RNaseT2* KO rat generation

*RNaseT2* KO rats were produced by SAGE Labs (now Horizon Discovery, St Louis, MO, USA) using CRISPR/Cas9-based technology. Briefly, single guide RNAs (sgRNAs) targeting the first and last exons were transcribed *in vitro* and validated in cultured rat C6 cells by Surveyor assay. The two active sgRNAs targeting 5′-CGGAGCCCGGGACAGCGCGATGG-3′ and 5′-GGGATGATGGTCTGCGAAGACGG-3′, respectively, along with *SpCas9* mRNA, were microinjected into pronuclei of fertilized eggs from Sprague Dawley rats. About 25-30 eggs were transplanted into each pseudopregnant female. Resulting live births were screened for the ∼17 kb large deletion by PCR and Sanger sequencing. The selected founders were backcrossed to WT rats to generate the heterozygous F1 generation. Rats were bred and studied at Alexion Pharmaceuticals (Lexington, MA, USA) or shipped to Northeastern University (Boston, MA, USA) for studies.

Rats were maintained on a 12:12 h light:dark cycle with a lights on at 07:00 h, and allowed access to food and water *ad libitum*. All rats were acquired and cared for in accordance with the guidelines published in the Guide for the Care and Use of Laboratory Animals (National Institutes of Health Publications No. 85–23, Revised 1985) and adhered to the National Institutes of Health and the American Association for Laboratory Animal Science guidelines. All animal work was performed in accordance with the approved animal protocols overseen by SAGE Labs, Alexion Pharmaceuticals and Northeastern University Institutional Animal Care and Use Committees.

### *RNaseT2* KO rat genotyping

Animals were genotyped by PCR with DNA isolated from tail samples using primers flanking the deletion site (5′-TCGGGGTCAAGTAAGTTTGG-3′ and 5′-AGGAACGCACAGTAGCACCTA-3′). PCR amplification was performed in a buffer containing 1× JumpStart Taq ReadyMix (Sigma-Aldrich, St Louis, MO, USA), 1 μM of each primer and 500 ng genomic DNA. After incubation at 95°C for 5 min, 35 cycles of 30 s at 95°C, 30 s at 60°C, 1 min at 68°C and a final incubation of 68°C at 5 min, the resulting mutant deletion allele produced a 496 bp product in line 12 animals. Alternatively, rat genotypes from tail biopsies were determined using real-time PCR with specific probes designed for the *RNaseT2* gene by a commercial vendor (Transnetyx, Cordova, TN, USA).

### Quantitative RT-PCR gene expression analysis

Rat brains were lysed with a BeadBug 3 Microtube Homogenizer with triple-pure molecular biology grade zirconium beads (Benchmark Scientific, Edison, NJ, USA). RNA was extracted from the tissue lysates using the Absolutely RNA Microprep Kit (Agilent Technologies, Santa Clara, CA, USA). cDNA was made using the SuperScript III Kit (Invitrogen, Waltham, MA, USA) and analyzed using Taqman Assays (Applied Biosystems, Waltham, MA, USA) for rat *RNaseT2* (Applied Biosystems, Rn01527359_g1) with a StepOnePlus Real-Time PCR System (Applied Biosystems) and software as per the manufacturer's recommendations. Rat *GAPDH* was used as an endogenous control for normalization (Applied Biosystems, Rn99999916_s1).

### Histology

Standard procedures were used to perform perfusions with PBS followed by 10% formalin. Adrenal glands, aorta, bladder, brain, cecum, colon, duodenum, epididymides, esophagus, eyes, femur, gastrocnemius, Harderian glands, heart, ileum, jejunum, kidneys, lacrimal glands, liver, lungs, mammary gland, mandibular lymph nodes, mesenteric lymph nodes, ovaries, pancreas, pituitary, prostate, rectum, salivary glands, sciatic nerve, seminal vesicle, skin, spinal cord, spleen, sternum, stomach, testes, thymus, thyroid, tongue, trachea, ureters and uterus were collected, fixed in 10% formalin, and transferred to 70% ethanol after 24 h. Tissues were trimmed routinely. Brains were trimmed coronally to yield six sections that included the following anatomic structures: olfactory bulbs; the cerebral cortex and the caudate-putamen; the cerebral cortex, thalamus and dorsal hippocampus; the mid-brain including the substantia nigra and ventral tegmental area; the cerebellum and pons; and the cerebellum and medulla oblongata. Tissues were processed for histopathology and embedded into paraffin blocks, sectioned at 5 μm, and stained with Hematoxylin and Eosin (H&E). Slides were analyzed by a Charles River board of certified veterinary pathologist with expertise in neurobiology in accordance with standard procedures (Charles River, Durham, NC, USA).

### Magnetic resonance imaging

#### Image acquisition

Animals were scanned at 300 MHz using a quadrature transmit/receive volume coil built into the rat head holder and restraining system for animal imaging (Animal Imaging Research, Holden, MA, USA). The design of the coil provided complete coverage of the brain from olfactory bulbs to brain stem with excellent B1 field homogeneity. Experiments were conducted using a Bruker Biospec 7.0T/20-cm USR horizontal magnet (Bruker, Billerica, MA, USA) and a 20-G/cm magnetic field gradient insert (ID=12 cm) capable of a 120-µs rise time (Bruker).

#### T1- and T2-weighted imaging and volumetric analysis

At the beginning of each imaging session, T1-weighted and T2-weighted high-resolution anatomical images were collected using the RARE pulse sequence. For T1-weighted images, the following parameters were used: 35 slice at 0.7 mm thickness; field of view (FOV) 3 cm; 256×256; repetition time (TR) 988 ms; echo time (TE) 9.45 ms; number of excitations (NEX) 6; 8 min 49 s acquisition time. T2-weighted images were collected with the following parameters: 35 slice at 0.7 mm thickness; FOV 3 cm; 256×256; TR 3900 ms; TE 48 ms; NEX 3; 6 min 14 s acquisition time.

To calculate brain volumes, we used a MRI Rat Brain Atlas (Ekam Solutions LLC, Boston, MA, USA) and registered the standard structural rat template image onto high-resolution T2-weighted images for each individual subject using a non-linear registration method implemented by the Unix-based software package Deformable Registration via Attribute Matching and Mutual-Saliency Weighting (DRAMMS) (https://www.cbica.upenn.edu/sbia/software/dramms/index.html). The atlas [image size 256×256×63 (h×w×d)] was then warped from the standard space into the subject image space (image size 256×256×40) using the deformation obtained from the above step using the nearest-neighbor interpolation method. For the volumetric analysis, each brain region was segmented and the volume values were extracted for all 174 ROIs (minus ventricle and white matter), calculated by multiplying the unit volume of voxel in mm^3^ by the number of voxels using in-house MATLAB scripts. To account for different brain sizes, ROI volumes were normalized by dividing each ROI volume by the total brain volume of that subject.

#### Diffusion-weighted imaging

DWI was acquired with a spin-echo echo-planar imaging (EPI) pulse sequence having the following parameters: TR/TE=500/20 ms, 8 EPI segments and 10 non-collinear gradient directions with a single b-value shell at 1000 s/mm^2^ and one image with a b-value of 0 s/mm^2^ (referred to as B0). Geometrical parameters were: 48 coronal slices, each 0.313 mm thick (brain volume) and with in-plane resolution of 0.313×0.313 mm^2^ (matrix size 96×96; FOV 30 mm^2^). The imaging protocol was repeated two times for signal averaging. Each DWI acquisition took 35 min and the entire MRI protocol lasted about 1 h 10 min.

Image analysis included DWI analysis of the DW-3D-EPI images to produce the maps of fractional anisotropy (FA) and radial diffusivity (RD). DWI analysis was implemented with Matlab^©^ (Mathworks, USA) and MedINRIA (1.9.0; http://www-sop.inria.fr/asclepios/software/MedINRIA/index.php) software. Because sporadic excessive breathing during DWI acquisition can lead to significant image motion artifacts that are apparent only in the slices sampled when motion occurred, each image (for each slice and each gradient direction) was screened, prior to DWI analysis, for motion artifacts; if found, acquisition points with motion artifacts were eliminated from analysis.

For statistical comparisons between rats, each brain volume was registered with the 3D rat atlas, allowing voxel- and region-based statistics. All image transformations and statistical analyses were carried out using in-house MIVA software (Zhang et al., 2003). For each rat, the B0 image was co-registered with the B0 template (using a 6-parameter rigid-body transformation). The co-registration parameters were then applied to the DWI indexed maps for the different indices of anisotropy [e.g. apparent diffusion coefficient (ADC), axial diffusivity (AD) and RD]. Normalization was performed on the maps since they provided the most detailed visualization of brain structures and allow for more accurate normalization. The normalization parameters were then applied to all DWI indexed maps. The normalized indexed map was smoothed with a 0.3-mm Gaussian kernel. To ensure that ADC, AD and RD values were not affected significantly by the pre-processing steps, the ‘nearest neighbor’ option was used following registration and normalization.

### Immunohistochemistry

For GFAP immunohistochemistry, tissue was cryosectioned to 40 μm and floating sections were incubated overnight at 4°C in an antibody to GFAP (mouse monoclonal anti-GFAP, Sigma-Aldrich, G3893) in a 1:1000 dilution under conditions including permeabilization with 0.3% Triton X-100 (Sigma-Aldrich, X-100), blocking with 1% bovine serum albumin (Sigma-Aldrich, A9647) and processing with the Vectastain Elite ABC HRP kit (Vector Laboratories, PK-6100, Burlingame, CA, USA). Tissue was then incubated for 1 h at room temperature in biotinylated horse anti-mouse IgG antibody, rat adsorbed, used in a 1:500 dilution (Vector Laboratories, BA-2001), then stained with SIGMAFAST™ 3,3′-diaminobenzidine tablets (Sigma-Aldrich, D4418).

### Behavioral tests

#### Novel recognition test

A novel recognition test (NOR) was used to assess episodic learning and memory-related stimulus recognition (Antunes and Biala, 2012; Bevins and Besheer, 2006). The apparatus consisted of a large black cube-shaped Plexi-glass box (L: 60.9, W: 69.2, H: 70.5 cm) with no lid that was indirectly dimly illuminated with two 40 W incandescent light bulbs. The task was performed over the course of 2 days. On day 1, animals were placed in the empty NOR box for an acclimation period of 15 min. The following day, for the first phase of testing (the familiar phase), two identical objects were placed in diagonal corners of the box 5 cm from each wall. Animals were placed in the box facing one of the two empty corners and allowed to investigate the objects over the course of 5 min. Animals were then placed back into their home cage for a period of 90 min. Animals were then exposed to the second phase of the test (the novel phase). During the novel phase, one of the familiar objects was replaced with a novel object and animals were placed in the box in one of the two empty corners and allowed to freely explore the objects for 3 min. All trials were analyzed for measures of object exploration during the novel object phase as described (Antunes and Biala, 2012). Exploration was defined as the rat directing its nose within 2 cm of the object or touching the object with the nose.

#### Barnes maze

The Barnes maze has been validated in assessing spatial learning and memory across various rodent models (Barnes, 1979; Fox et al., 1998; Harrison et al., 2009). The maze consists of a circular platform (121 cm in diameter, elevated 40 cm) with 18 escape holes along the perimeter at 30 cm intervals. A black, removable enclosed Plexiglas goal box was positioned under a single escape hole on the underside of the maze (L: 40.0, W: 12.7, H: 7.6 cm) in the same position relative to the testing room across all trials. Between trials, the maze was rotated 45° and the goal box shifted accordingly for cardinal consistency. Each trial began with animals being placed inside the goal box for 1 min and then under an enclosed container at the center of the maze for 30 s, that was then lifted to start the trial. If animals did not find the goal box within the test period (4 min), they were gently nudged into the box and allowed to stay for 1 min, and then placed back in their home cages between trials (three trials/day for 4 days). For both the NOR and the Barnes maze, all trials were video recorded and analyzed using manual methods by experimenters blind to treatment condition and verified with automated scoring using ANY-maze^®^ software (Stoelting, Wood Dale, IL, USA).

#### Beam walk

A tapered balance beam equipped with sensors capable of detecting foot faults on either side of the beam (Dragonfly Inc., Ridgeley, WV, USA) was used to assess fine-motor coordination as previously described (Shear et al., 2010; Williams et al., 2005). Briefly, the beam was 150 cm long and started at a width of 5.5 cm, and tapered down to 1.5 cm immediately prior to an opening that led into an enclosed goal box. The beam was elevated 120 cm off the ground and fitted with a safety hammock approximately 80 cm below the beam in case the animals fell off. On either side of the beam, approximately 4 cm below the ledge of the beam surface, there were two 2 cm wide ledges that ran the length of the beam that were connected to sensors that recorded the number of times each animal's paws slipped off the beam surface (‘foot faults’). The sensor ledges were divided along the length of the beam into three separate sections (approximately 47 cm each) on either side that recorded faults at the beginning (the widest portion of the beam), the middle (the intermediate portion of the beam) and the end (the thinnest portion of the beam).

All animals were acclimated to the beam over 2 days and exposed to three training trials each day. For training, animals were initially placed inside the goal box and allowed to acclimate for 60 s. Animals were then placed on a start platform illuminated by a single white light (60 W incandescent light bulb) to serve as a mildly aversive stimulus to motivate the animal to traverse the beam and enter the goal box. Once the animals reached the goal box, they remained there for 60 s and were then placed back in their home cage until the next trial. The day after training, animals were tested over three trials per day for 2 days. Testing conditions were similar to training conditions with the aversive light stimuli removed. Foot faults and goal box latency were recorded for each trial and averaged for each day.

#### Rotarod

The rotarod test isolates equilibrium and motor behavior using a cylindrical tube 4 cm in diameter rotating at an increasing frequency (Hamm et al., 1994). Animals were acclimated to the rod over the course of two consecutive days, with three trials per day. For training, animals were placed on the rod rotating at a frequency of 5 rpm for 3 min. If, at any time, the animals fell off, they were immediately returned to the rod surface for the remainder of the training period. The day following training, animals were tested for two consecutive days with three trials per day. During testing trials, animals were placed on the rod set at a frequency of 1 rpm increasing linearly at a 1.7 cm/s^2^ acceleration rate for a total of 210 s ending at a maximum frequency of 50 rpm. Latency to fall off the rod was recorded and averaged across trials and days.

### Lysosome analysis

The activities of NAG and acid phosphatase enzymes in hippocampal sections were measured using colorimetric analysis with commercial kits available from Abcam and Biovision, respectively. The LAMP1, LAMP2, NAG and acid phosphatase gene expression levels were measured using qPCR as described previously (Trivedi et al., 2017). Protein expression was evaluated using western blot as previously described (Talekar et al., 2016). Briefly, proteins were extracted from hippocampal sections using Total Protein Extraction Kit (Millipore, Billerica, MA, USA) and Powergen 125 tissue homogenizer (Fisher Scientific, Waltham, MA, USA). Tissue lysate samples were analyzed for total protein concentration using the BCA assay (Pierce, Rockford, IL, USA). Total protein extract (50 μg) was run on a precast 4-20% sodium dodecyl sulfate-polyacrylamide gel electrophoresis system at 200 V for 30 min. Subsequently, protein bands on the gel were transferred onto a polyvinylidene difluoride membrane by an iBlot Dry Blotting System (Invitrogen). The membrane was blocked with 5% milk in Tween-containing Tris-buffered saline (TBST) for 1 h at room temperature. Membrane was cut and incubated with 1:1000 dilution of primary rabbit anti-GAPDH antibody (Abcam, ab9485, Cambridge, MA, USA) or 1:1000 dilution of primary rabbit monoclonal anti-LAMP1 antibody (Abcam, ab62562) or 1:1000 dilution of primary rabbit monoclonal anti-LAMP2A antibody (Abcam, ab125068) or 1:1000 dilution of primary rabbit polyclonal to LC3B (Abcam, ab48394) or 1:1000 dilution of primary rabbit monoclonal to p62/SQSTM1 (Abcam, ab109012) separately overnight at 4°C. Membranes were then washed three times with TBST and incubated with 1:2000 dilutions of secondary anti-rabbit horseradish-peroxidase-conjugated IgG (Abcam, ab6721) in TBST for 1 h at room temperature. After rinsing excess antibody with TBST and water, 4 ml ECL substrate (Pierce) was added and mixed with membranes for 5 min, which is cleaved by peroxidase to give a chemiluminescent product. The membranes were visualized using a Kodak Digital X-ray Specimen System. GAPDH was used as a protein-loading control. Quantification was performed using ImageJ software, and ratios were calculated respective to the GAPDH concentrations and normalized to WT.

### Statistics

Unpaired two-tailed Student's *t*-tests or analysis of variance (ANOVA) were performed unless otherwise noted. Single-sample *t*-tests were used during NOR testing to determine if novel object preference occurred due to chance (theoretical mean set at 0.5). GraphPad Prism (GraphPad Software, La Jolla, CA, USA) was used for graphing and statistical analyses. All experiments were performed independently at least three times and pooled data is represented by the mean and standard deviation (s.d.) or standard error of the mean (s.e.m.) as noted.

## Supplementary Material

Supplementary information
