# Supplementary material for: RNaseT2 knockout rats exhibit hippocampal neuropathology and deficits in memory
Source: Dis Model Mech. 2018 Jun 27;11(6):dmm032631. doi: 10.1242/dmm.032631 (PMC6031352; doi:10.1242/dmm.032631)
Supplement: Supplementary information [file dmm-11-032631-s1.pdf]

SUPPLEMENTARY MATERIAL

Fig. S1. Rat RNASET2 CRISPR/Cas9 knockout design strategy and deletion screen. (A) Two pairs of sgRNAs (gRNA4, gRNA5) were designed to delete the 9 exons of *RNaseT2*. Primers flanking each sgRNA site were designed to test individual NHEJ activity and screen for deletion mutations between the two target sites (red arrows). (B) 20 founder animals were screened for the ~17kb deletion using the F1/R2 primers described in A. Lines 11, 12, 15, 19, and 20 contained the mutant band. The bands were gel purified and Sanger sequence verified. Lines 12, 19, and 20 contained a 16937 bp deletion, line 15 contained a 16945 bp deletion, and line 11 contained a 17109 bp deletion and 35 bp insertion. Lines 11, 12, and 19 were backcrossed to wild type animals to generate F1 heterozygous progeny. (wt: wild type negative control, (+): cell lysate positive control, NTC: no template negative control) (C) qPCR analysis of line 11, 12, and 19 brain *RNaseT2* expression of wild type (WT) and homozygous (KO) rats. n=6. Vertical bars denote SEM. An unpaired two-tailed Student's t test was performed. *P=0.0028.

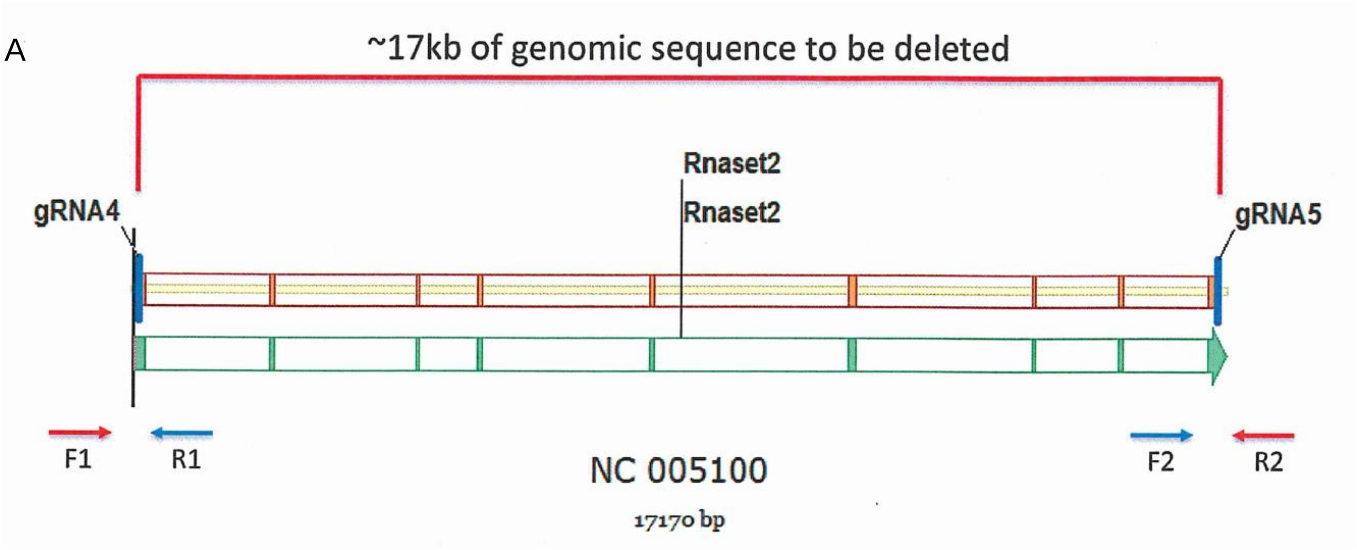
A
~17kb of genomic sequence to be deleted
Rnaset2
Rnaset2
gRNA4
gRNA5
F1
R1
F2
R2
NC 005100
17170 bp

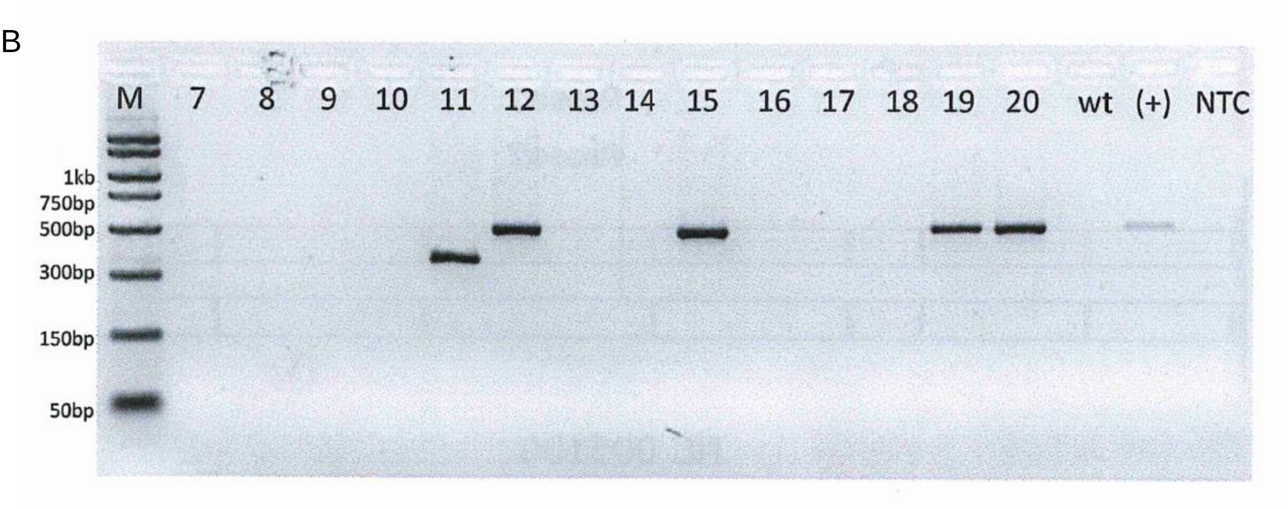
B
M
7
8
9
10
11
12
13
14
15
16
17
18
19
20
wt
(+)
NTC
1kb
750bp
500bp
300bp
150bp
50bp

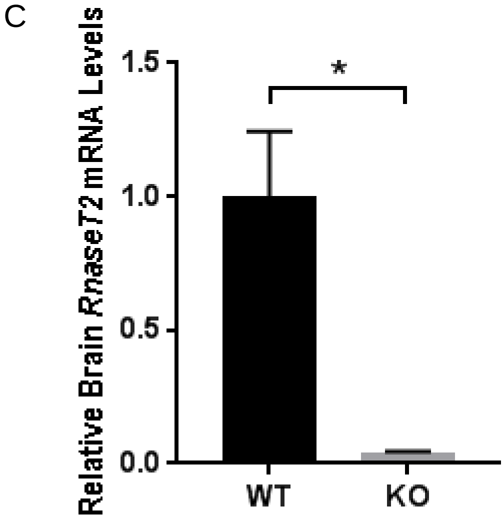
C
Relative Brain *Rnase T2* mRNA Levels
1.5
1.0
0.5
0.0
*
WT
KO

Table S1

Click here to Download Table S1

Table S2

Click here to Download Table S2

Table S3

Click here to Download Table S3
